# Compositional Evolution of Individual CoNPs on Co/TiO_2_ during CO and Syngas Treatment Resolved through Soft XAS/X-PEEM

**DOI:** 10.1021/acscatal.3c03214

**Published:** 2023-11-28

**Authors:** Chengwu Qiu, Yaroslav Odarchenko, Qingwei Meng, Hongyang Dong, Ines Lezcano Gonzalez, Monik Panchal, Paul Olalde-Velasco, Francesco Maccherozzi, Laura Zanetti-Domingues, Marisa L. Martin-Fernandez, Andrew M. Beale

**Affiliations:** † Department of Chemistry, University College London, 20 Gordon Street, London WC1H 0AJ, U.K.; ‡ Research Complex at Harwell (RCaH), Harwell, Didcot, Oxfordshire OX11 0FA, U.K.; § School of Chemical Engineering and Light Industry, Guangdong University of Technology, Guangzhou 510006 (China); ∥ Diamond Light Source, Harwell, Didcot, Oxfordshire OX11 0DE, U.K.

**Keywords:** redox state, FTS catalysts, CoNPs, soft XAS-X-PEEM, syngas

## Abstract

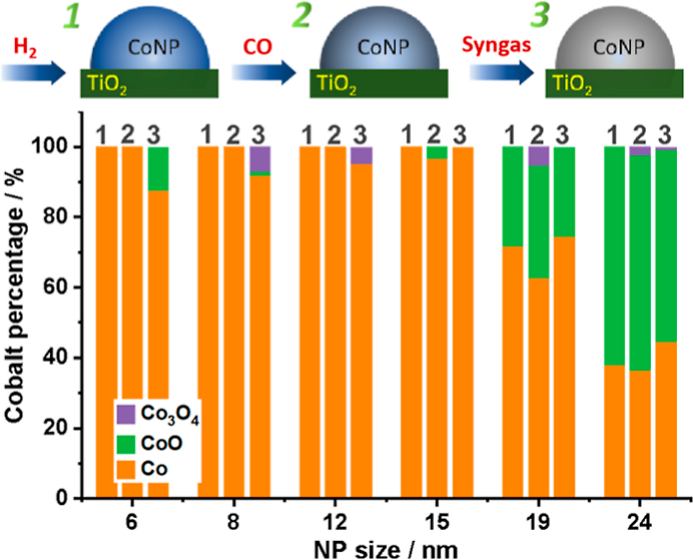

The nanoparticle (NP) redox state is an important parameter in the performance of cobalt-based Fischer–Tropsch synthesis (FTS) catalysts. Here, the compositional evolution of individual CoNPs (6–24 nm) in terms of the oxide vs metallic state was investigated in situ during CO/syngas treatment using spatially resolved X-ray absorption spectroscopy (XAS)/X-ray photoemission electron microscopy (X-PEEM). It was observed that in the presence of CO, smaller CoNPs (i.e., ≤12 nm in size) remained in the metallic state, whereas NPs ≥ 15 nm became partially oxidized, suggesting that the latter were more readily able to dissociate CO. In contrast, in the presence of syngas, the oxide content of NPs ≥ 15 nm reduced, while it increased in quantity in the smaller NPs; this reoxidation that occurs primarily at the surface proved to be temporary, reforming the reduced state during subsequent UHV annealing. O K-edge measurements revealed that a key parameter mitigating the redox behavior of the CoNPs were proximate oxygen vacancies (O_vac_). These results demonstrate the differences in the reducibility and the reactivity of Co NP size on a Co/TiO_2_ catalyst and the effect O_vac_ have on these properties, therefore yielding a better understanding of the physicochemical properties of this popular choice of FTS catalysts.

## 1 Introduction

Fischer–Tropsch synthesis (FTS) is a catalytic process used to convert syngas (CO + H_2_) to hydrocarbons and eventually to fine chemicals and liquid fuels.^1,2^ An active and stable catalyst with a selectivity toward heavier hydrocarbons is typically desired and normally achieved by supporting CoNPs on a solid oxide support.^1^ Titania (TiO_2_), due to its moderate specific area, high thermal and chemical stability, and amphoteric properties, is considered perhaps the most appealing support.^3–5^ Furthermore, the metal-support interaction between the CoNPs and the solid oxide can be considered strong enough to allow for striking a balance in terms of reducibility, dispersion, and stability.^3^ As a result, Co/TiO_2_ catalysts have proven to be particularly attractive for the production of long-chain hydrocarbons, i.e., waxes.^6,7^ Despite this suitability, Co/TiO_2_ is not immune to deactivation and is known to become inhibited by phenomena such as the blockage of active sites and solid-state reaction, and as such, this loss of FTS activity makes the understanding of CoNP evolution, particularly supported on TiO_2_, a topic of great interest.^8^

CoNPs in FTS catalysts are known to undergo physicochemical changes during activation and under reaction conditions. These include changes in morphology^9–13^ and composition,^11,14–16^ both of which can affect the FTS performance, the significance and extent of which are affected by parameters such as preparation method, catalyst composition, activation procedures used, and the reaction conditions.^3,11,17–19^ One of the more readily investigated phenomena concerns the (re)oxidation of CoNPs and how this shows a dependency on both the support type and the particle size of the CoNPs. Indeed CoNP size effects on FTS activity and product selectivity have been well-researched and a critical size range of 5–10 nm was determined.^20–23^ In contrast, fewer studies have been reported on the stability of a particular oxidation state of Co as a function of particle size.^24–26^ Some specific reports have shown that, for example, CoNPs with a particle size of <7 nm, supported on SiO_2_ or Al_2_O_3_ were easy to reoxidize and/or form metal-support compounds.^24,27,28^ More recently, it was also observed that on Al_2_O_3_, more surface carbon accrues with increasing CoNP size during the FTS reaction^26^ and, furthermore, that a size-dependent Kirkendall effect operates during treatment of CoNPs in an oxidizing environment.^29^ Interaction of Co with the oxide support also can affect the reducibility or stability of the cobalt oxide (CoO_*x*_) particles. A CoO_*x*_ particle of the same size may have different stability depending on the support oxide on which it is located.^30–32^ There is surprisingly sparse information on the relationship between CoNP size and its redox state under relevant reactive gas environments, despite the well-documented benefits of utilizing TiO_2_-supported CoNP as an FTS catalyst. As such, it is useful to examine this size-dependent component evolution for TiO_2_-supported CoNPs.

One of the challenges when determining the physicochemical properties of supported CoNPs is that the majority of studies performed concern powdered forms of the catalyst comprising a range of CoNP sizes, which, although insightful,^24,26–28^ provide results that are an average of the whole system where specific size effects are difficult to deconvolute. In this regard, investigating the component changes in an individual NP is far more revealing for understanding the size-dependent evolution of CoNPs in various gas atmospheres. X-ray absorption spectroscopy/X-ray photoemission electron microscopy (XAS/X-PEEM) is a powerful technique combination that enables chemical and structural changes on the surface of a sample or catalyst to be followed under defined chemical (reaction) conditions.^33^ The technique, first developed by Ernst Brüche in the early 1930s,^33,34^ allows for obtaining not only spatially resolved images of NPs with a spatial resolution approaching the 10s of nm but also allowing the determination of the chemical composition, namely, their oxidation and coordination states, by recording images of objects under illumination with an energy-tunable X-ray source. Some of this work has concentrated on surface-sensitive reactions over large domains (μm scale),^33,35,36^ although more recently individual NPs supported on (semi)conductor (silicon, GaAs) substrates have also been studied.^37–40^ Research performed on pure oxide substrates, i.e., SiO_2_ or Al_2_O_3_, seldom exists due to their unsuitability (their low conductivity), but since TiO_2_ is a semiconductor it is ideally suited for this study.^9^ Indeed, we have shown in previous work that the XAS/X-PEEM technique is well-suited for characterizing the size-dependent redox state of individual variously sized CoNPs on TiO_2_ single-crystal substrates after various gas treatments, and therefore, this technique is well-suited for the research reported here.^41^

In this work, we probe the compositional evolution of individual CoNPs during the CO/syngas treatment using XAS/X-PEEM. A two-dimensional (2D) catalyst comprising a rutile TiO_2_ (110) substrate on which Co_3_O_4_ NPs of various sizes were dispersed (6–24 nm) was used as a model system. By analyzing the XAS spectra, the component evolution of individual CoNPs during CO and syngas treatment at various temperatures was examined. It was observed that CO itself can only dissociate on large NPs (≥15 nm), leading to oxidation of these NPs but did not affect the redox state of cobalt of the smaller NPs (<15 nm). In contrast, during syngas dosing, CO dissociation with the assistance of H_2_ can occur on small NPs (≤12 nm) leading to their reoxidation; however, syngas also induces further reduction in the bigger NPs (>15 nm).

## 2 Results and Discussion

### 2.1 Before and after H_2_ Treatment

**Figure 1:**
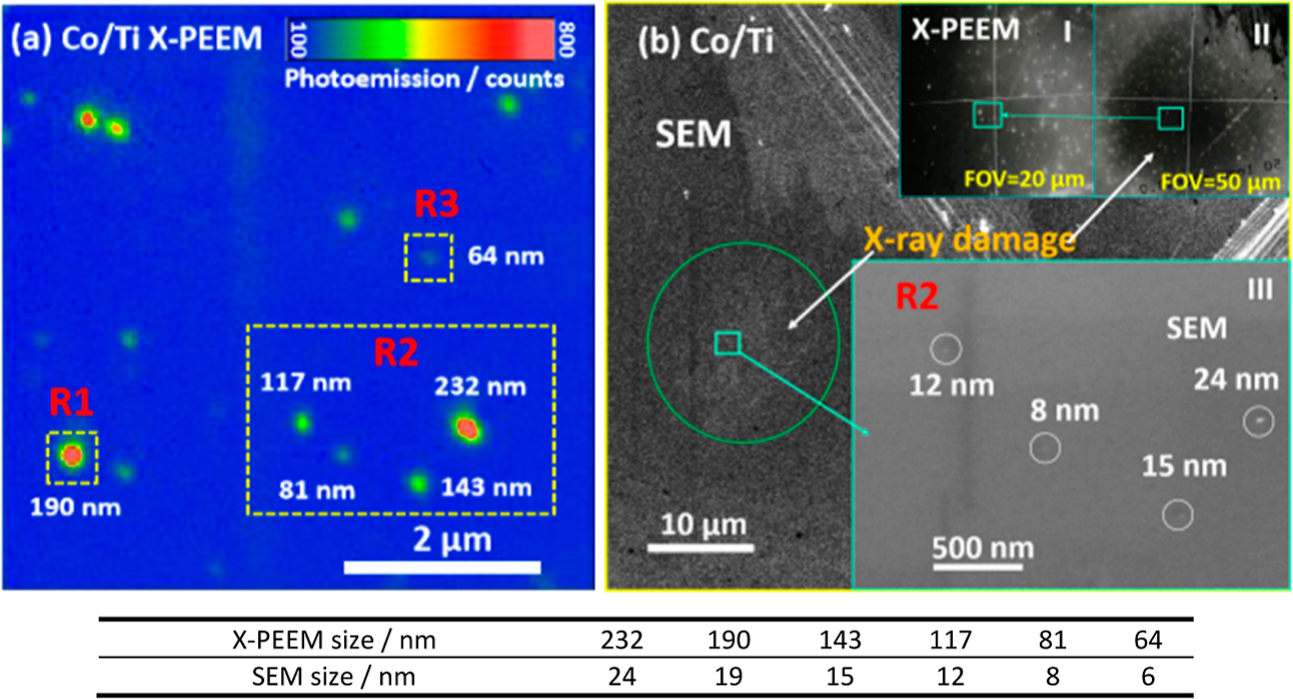
Correlation of the size of individual CoNPs in X-PEEM (a) recorded at 778.4 eV and (b) in high-resolution SEM, the absolute size of which is 10 times smaller than the size observed in X-PEEM images. Inserts in (b): (I–II) X-PEEM images at 20 and 50 μm FOV; (III) SEM image corresponding to region R2 in (a). FOV: Field of view.

The Co/TiO_2_ sample was loaded into the X-PEEM chamber to view individual CoNPs (Figure 1a). These particles were readily identified on the surface by round spots with different levels of brightness and diameters, reflecting the differences in size of the NPs. After the X-PEEM experiment, the absolute size (diameter) of the corresponding CoNPs was confirmed by high-resolution SEM (Figure 1b) and estimated to be ∼10 times smaller than the diameter observed in the X-PEEM images. The discrepancy is due to the limitations of spatial resolution (theoretical ∼10 nm) of the X-PEEM technique, in combination with the conductivity of the semiconductor rutile and topography of the sample.^38,42,43^ The presynthesized Co_3_O_4_ NPs (6, 11, and 18 nm in size, see the XRD patterns in Figure S1) for the 2D Co/TiO_2_ preparation were initially mixed before application, details of which can be found in the Supporting Information.

**Figure 2:**
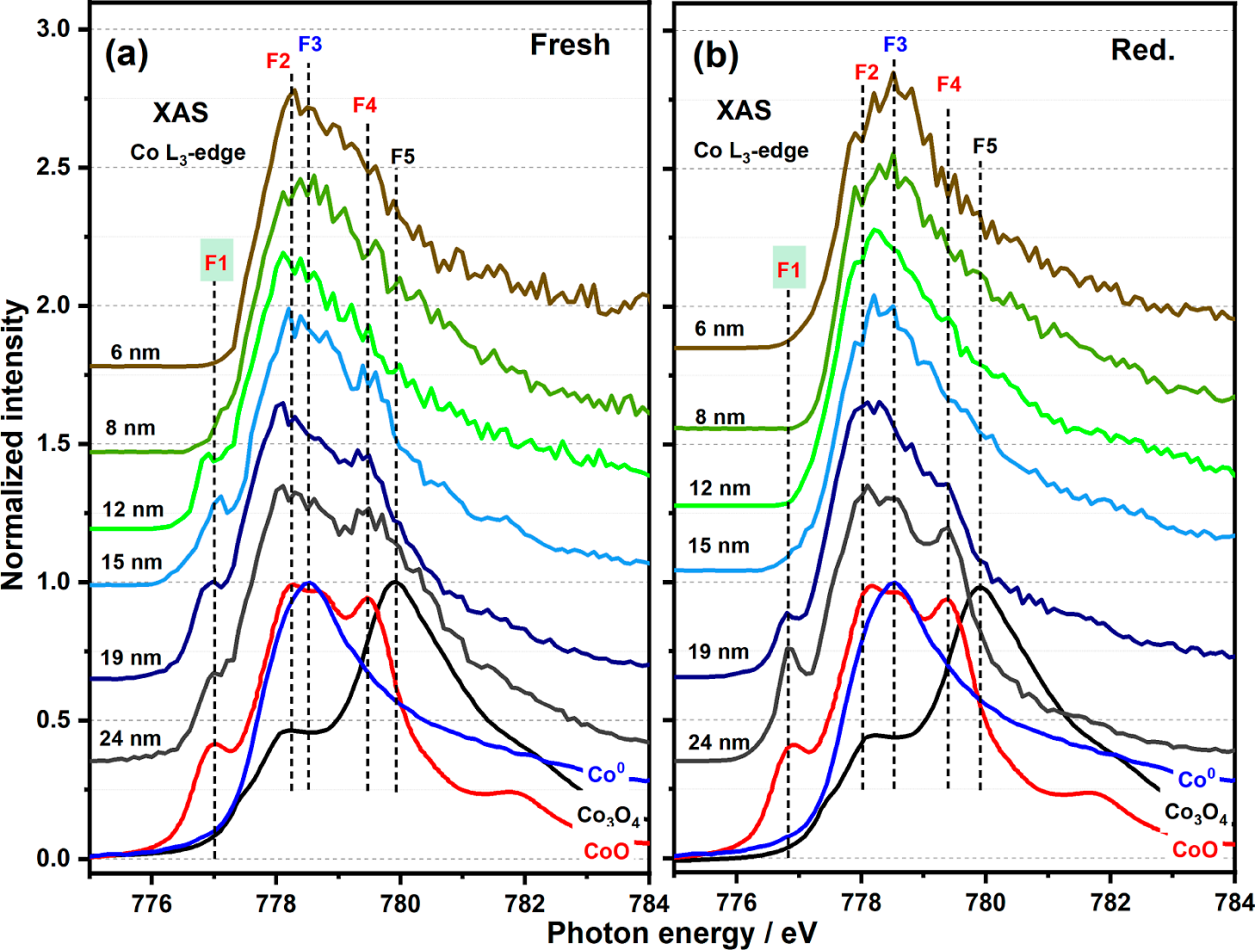
XAS spectra of the Co L_3_-edge in fresh (a) and 623 K H_2_ reduced (b) Co/TiO_2_ 2D catalysts changing with CoNP sizes. The spectra were recorded from the center of the NPs. The reported sizes of the NPs are those determined by SEM. NPs ≤8 nm can be fully transformed to metallic cobalt before H_2_ reduction, while those ≤15 nm after H_2_ reduction. F1, F2, and F4 indicate the key features in the reference spectra for CoO; F3 for metallic Co^0^ and F5 for Co_3_O_4_.

**Figure 3:**
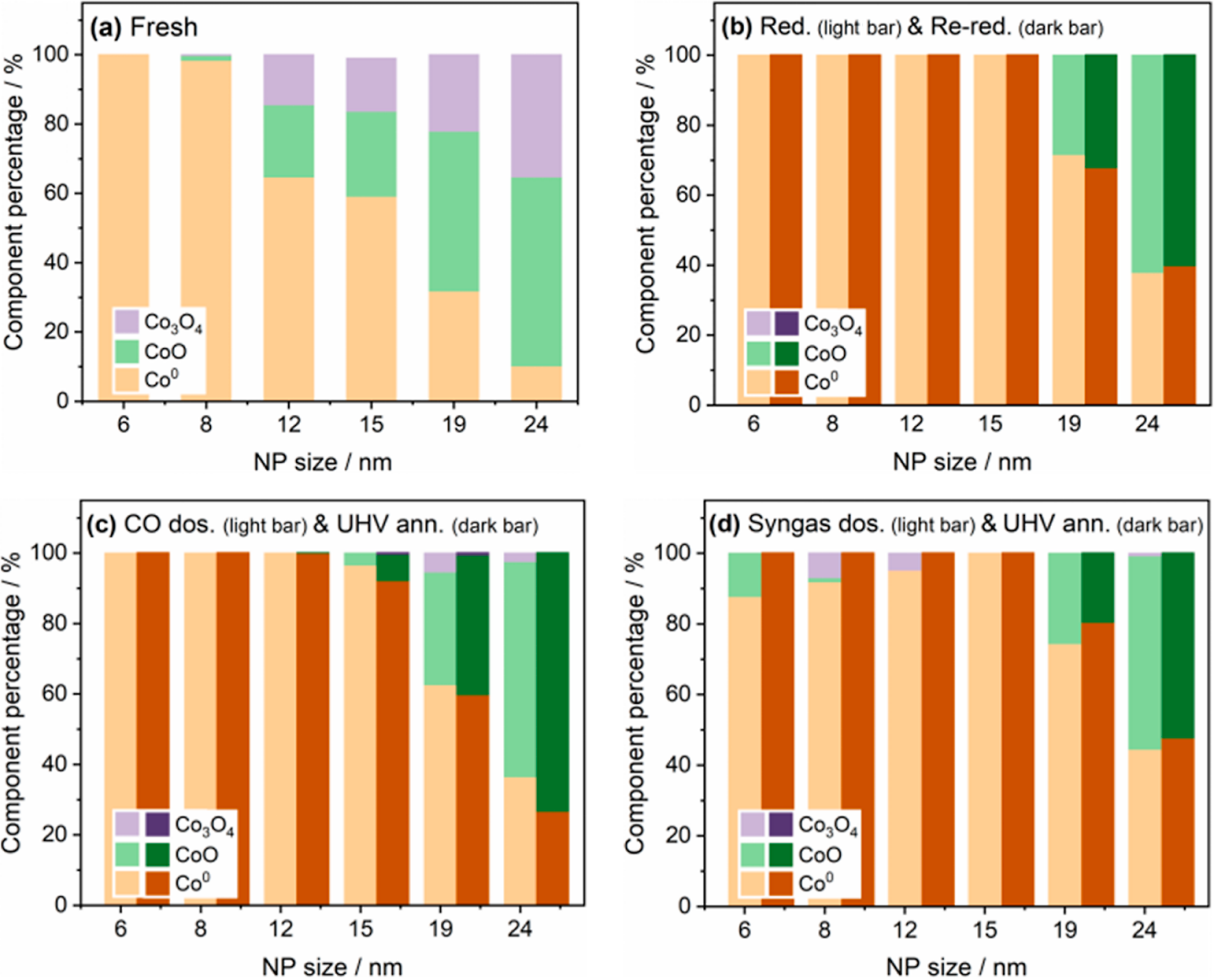
Linear combination fitting results of XAS spectra of the Co L_3_-edge (775–784 eV) in treatments.

The fresh and reduced cobalt XAS spectra of the focused NPs (i.e., recorded from the middle of the NPs) are displayed in Figure 2. Note that the size-labeling of the NPs has been determined from the SEM images. In Figure 2a, according to their XAS spectra, all the “fresh” NPs are not present as pure Co_3_O_4_ when compared with the reference standard. This is confirmed by the linear combination fitting results for those NPs shown in Figures 3 and S2 and Table S1. We observe that even in the fresh sample, smaller NPs (i.e., 8 and 6 nm) were reduced to CoO and even further to Co^0^. XAS spectra recorded on the sample after 623 K H_2_ (3 h) treatment revealed the presence of a greater degree of metallic cobalt, with Figure 2b showing that small NPs (≤15 nm) were fully reduced to metallic cobalt, while bigger NPs (i.e., 19 and 24 nm) comprised a mixture of CoO and Co^0^ (Figure 3). Analyzing the O K-edge XAS spectra for the NPs (Figures S3a, S6–S8, and Figure 5, acquired on the NPs), we observe relatively lower intensities of the 3d t_2g_ than the e_g_ resonance bands in addition to a more narrow splitting between the centroid position of these two bands, i.e., ∼2.3 eV (10Dq). Note that in the rutile standard, the intensity of the t_2g_ resonance band is higher than the e_g_ while the splitting or 10Dq is about 2.7 eV^44^ (Figure 5d). This was determined previously to be caused by the presence of oxygen vacancies (O_vac_) in the TiO_2_ and is particularly noticeable at the perimeter of the NPs.^44,45^ Those oxygen vacancies are thought to form during the air plasma treatment and have been reported to have promotional effects on cobalt oxide reduction.^46–48^ From the corresponding XPS spectra (O 1s and Ti 2p, Figure S3b,c), the number of O_vac_ (apart from ∼73% of Ti–O–Ti and ∼15% Ti–OH) on the titania surface is determined to be around 12%. Because of the higher surface-to-bulk ratio for the smaller CoNPs, they are easier to reduce.

**Figure 4:**
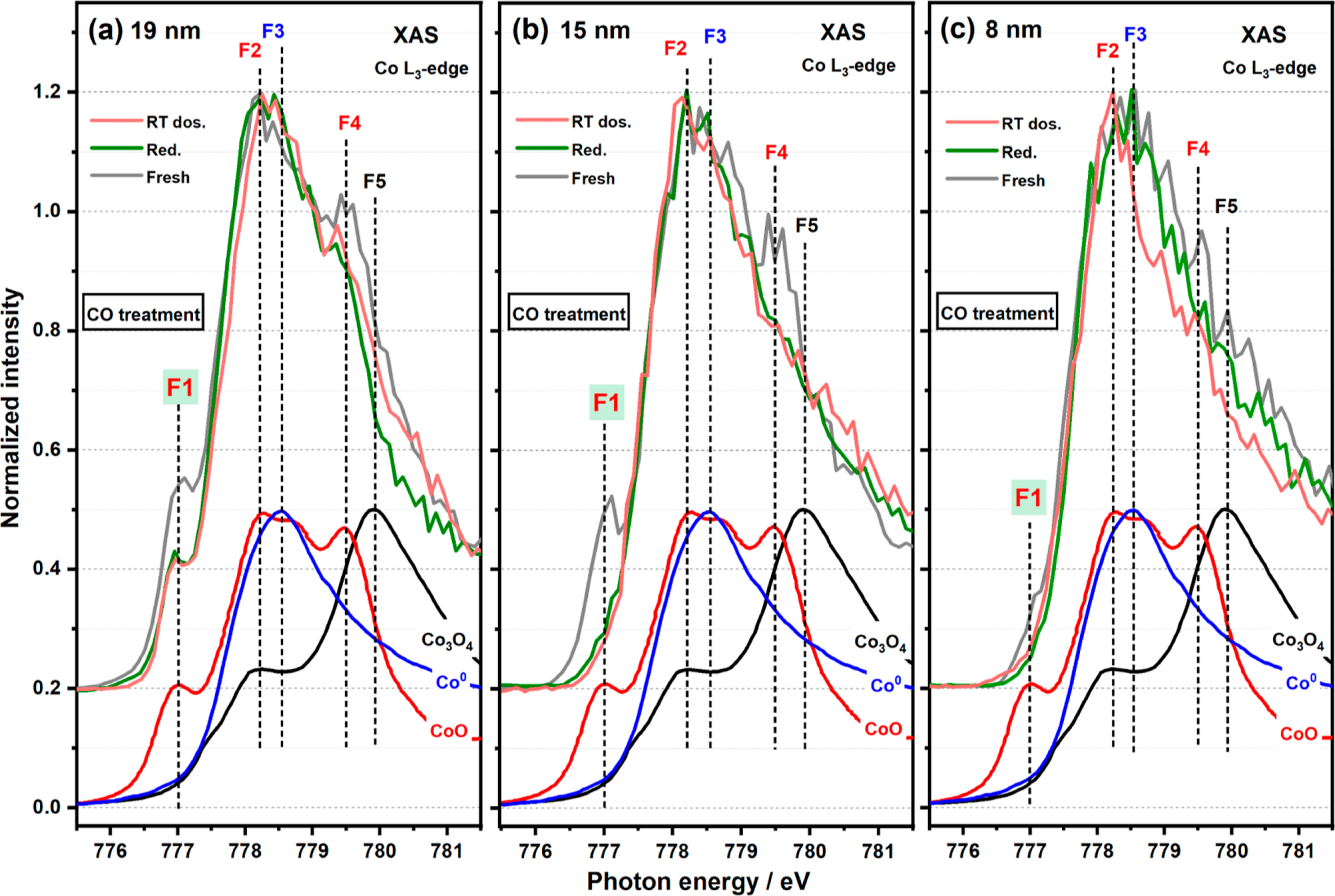
Co L_3_-edge XAS spectra of 19 (a), 15 (b), and 8 nm (c) CoNPs in CO treatment. CO was dissociated at room temperature in big NPs (≥15 nm), leading to reoxidation of those NPs, while it remained undissociated on small NPs.

**Figure 5:**
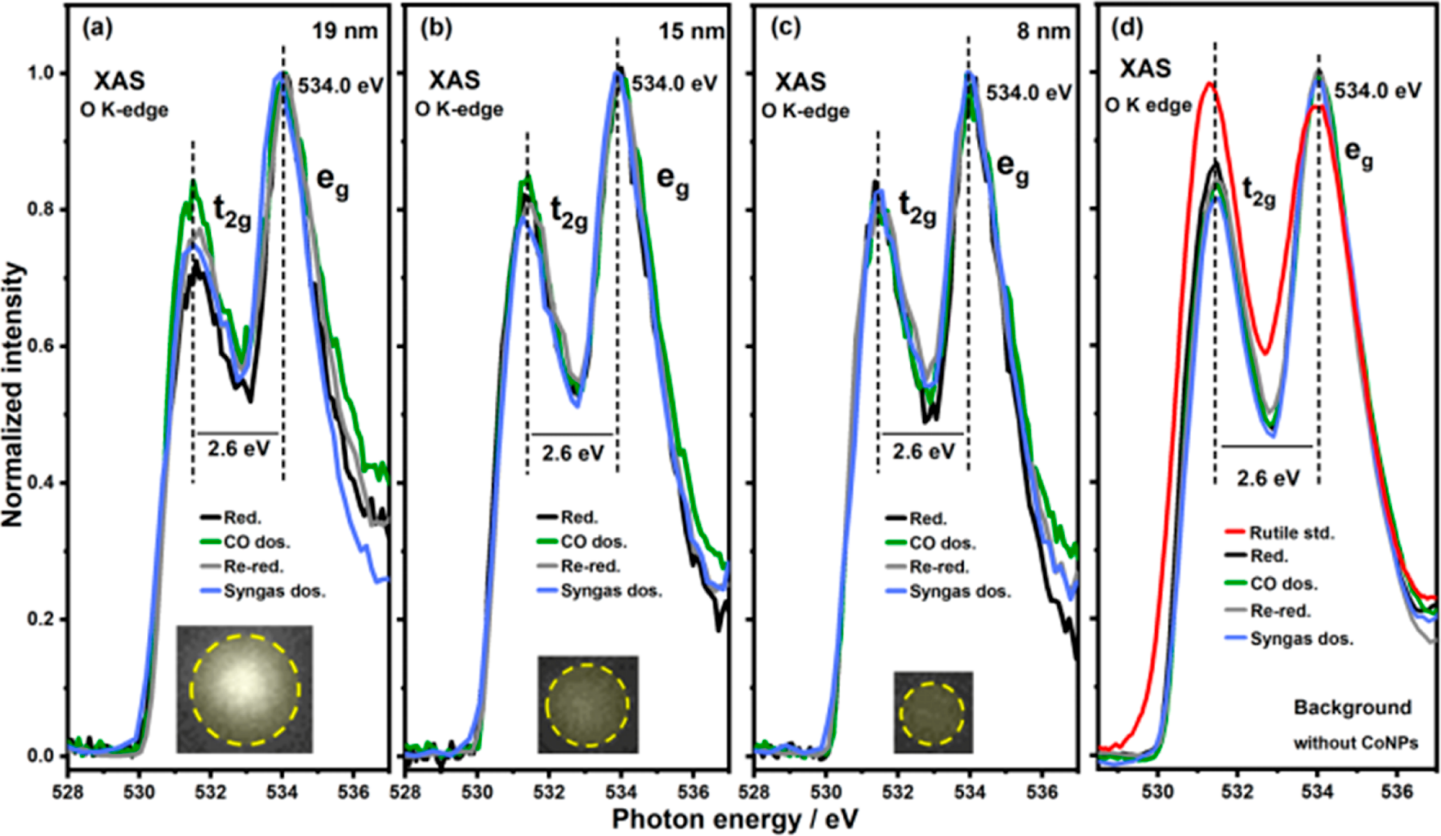
O K-edge XAS spectra of (a) 19, (b) 15, (c) 8 nm CoNPs, and (d) background titania substrate after gas-dosing with H_2_, CO, and syngas. All of the spectra are normalized to 1 via the intensity of the transition into the empty e_g_ orbitals. The intensity of the transition to the t_2g_ orbitals for the samples is always lower than that seen in rutile, [red line, panel (d)] and the transition to the e_g_ orbitals in addition to a lower separation/splitting energy (10Dq ∼ 2.7 eV in standard rutile) is due to the presence of O_vac_ and Ti^3+^ species.

### 2.2 CO Treatment

The reduced NPs were exposed to pure CO gas (1 × 10^–6^ mbar, 30 min) in a prechamber at room temperature and then introduced into the main chamber, and XAS spectra were acquired. The spectra recorded at the Co L_3_-edge are shown in Figure 4, S4, and S5. On initial examination of the XAS spectra, there appear to be minimal differences between all the NPs before and after CO adsorption. However, from linear combination fitting data, the results of which are given in Figure 3, larger NPs (≥15 nm) were observed to partially oxidize, reforming both CoO and Co_3_O_4_ after annealing (ann.) of this sample in the ultrahigh vacuum (UHV) X-PEEM chamber (1 × 10^–9^ mbar, 30 min) at 493 K. This can be seen by virtue of noticeable increases in the F1 feature (∼777 eV) in Figures S4a,b and S5a. In contrast, no detectable change in oxidation state was observed in small NPs (<15 nm) (Figure 3c). Previous work by Tuxen et al. has demonstrated that the difference in Co state is related to the ability of the CoNPs to dissociate CO, seemingly larger particles producing C* and O* that oxidize the Co on contact.^49^ In contrast, CO is thought to adsorb on Co while being retained in molecular form on smaller NPs, although this will leave the Co L_3_-edge spectra otherwise unchanged (as per the spectra of the reduced or metallic cobalt) even when adsorbing molecular CO.^49^ It is interesting that for the 15 nm CoNP, no Co_3_O_4_ was observed to reform during CO adsorption, with only CoO detected in Figure 3c, which is consistent to previous observations.^50^

To further confirm this observation, we analyzed the changes of O K-edge spectra on the NPs (Figures 5 and S6–S8), although the contribution of the cobalt oxide from the bigger NPs (19/24 nm) will obfuscate an accurate determination of the changes in the spectra.^51^ Note the main features in the O K-edge XAS spectra are labeled accordingly as t_2g_ and e_g_ due to their origination as transitions from O 1s to unoccupied O 2p–Ti 3d orbitals in an O_h_ crystal field splitting at 531.5 eV (t_2g_) and 534.0 eV (e_g_), respectively. Such features are consistent with the presence of the rutile polymorph.^52,53^ Here we utilize the differences in the normalized relative intensity of these features to provide insights into the local structural and electronic state of Ti. For example, the relative decrease in the t_2g_ peak intensity and 10Dq has previously been ascribed to Ti^3+^ formation and the increased electron population in the Ti 3d t_2g_ state, reducing the dipole transition probability from the O 1s orbital.^44,45,54,55^ The number of surface O_vac_ or Ti^3+^ can be correlated with 10Dq and ratio of *I*_eg_/*I*_t2g_, namely, the lower the 10Dq or higher the *I*_eg_/*I*_t2g_, the more O_vac_ or Ti^3+^ is present on the surface.

Specifically, we observe increased intensities of the t_2g_ peaks in the spectra from NPs ≥15 nm, particularly from the spectra of the 19 nm NP (an increase of 15% in Figure 4a) after CO dosing (dos.), which indicates a loss of O_vac_ and reformation of TiO_2._ We propose that the O species used to “fill in” the O_vac_ originate from the dissociated CO on CoNPs. CO dissociation at room temperature has previously been attributed to the shortage of adsorbed H (the sample was annealed in UHV to remove surface H prior to CO dosing) and its higher dissociation energy at this temperature.^49^

Of course, these O species can also oxidize cobalt metal; however, the O in CoNPs can be captured by nearby O_vac_, leading to the reduction of the oxidized NPs. This is supported by our previous work where we observed that the electrode potential between the Ti^3+^ and Ti^4+^ when compared to Co^2+^ and Co metal redox pair acts as a driving force for this behavior.^41^ Note that a relatively small increase of the t_2g_ (by 2.9%) in the 24 nm NP (Figure S8a) was also due to a large amount of cobalt oxide (>60%) present in this NP and we suspect therefore that these NPs are less able to dissociate CO. In contrast, the relatively unchanged O K-edge (t_2g_ peaks) in Figures 5c, S6c, and S7b,c indicates that the <12 nm-sized particles remain reduced even after thermal annealing in UHV, indicating a lack of CO dissociation on the smaller NPs. We should remark at this stage that the O K-edge spectra are dominated by the behavior of the largest component (rutile TiO_2_) see Figures S4d and S6d and hence disentangling the contribution of cobalt oxide (∼531 eV) and adsorbed molecular CO (∼534 eV) was not possible.^49^ As such, the interpretation of the changes in O K-edge data is largely comparative and inspired by our previous observations, where O_vac_ on rutile were shown to diminish as NPs of cobalt oxide in the vicinity reduced or as we propose here, CO dissociation occurs.^41^ It should be noted, however, that the intensity of the t_2g_ transition in the sample never matches that seen in crystalline rutile suggesting that the O_vac_ persist in the sample even in the presence of CO.

From these data, the critical size of CoNPs for CO dissociation at room temperature is proposed to be 12 nm, as evidenced by the changes (or lack thereof) of the cobalt composition as shown in Figure 3 together with the unchanged t_2g_ peak intensity from the spectrum of the 12 nm particle (Figure S8b). Other data indicated that this particle size represents something of a watershed with the O K-edge and Ti L_3_-edge spectra undergoing little change after CO dosing at room temperature (Figures S6, S7, and S9).

### 2.3 Syngas Treatment

**Figure 6:**
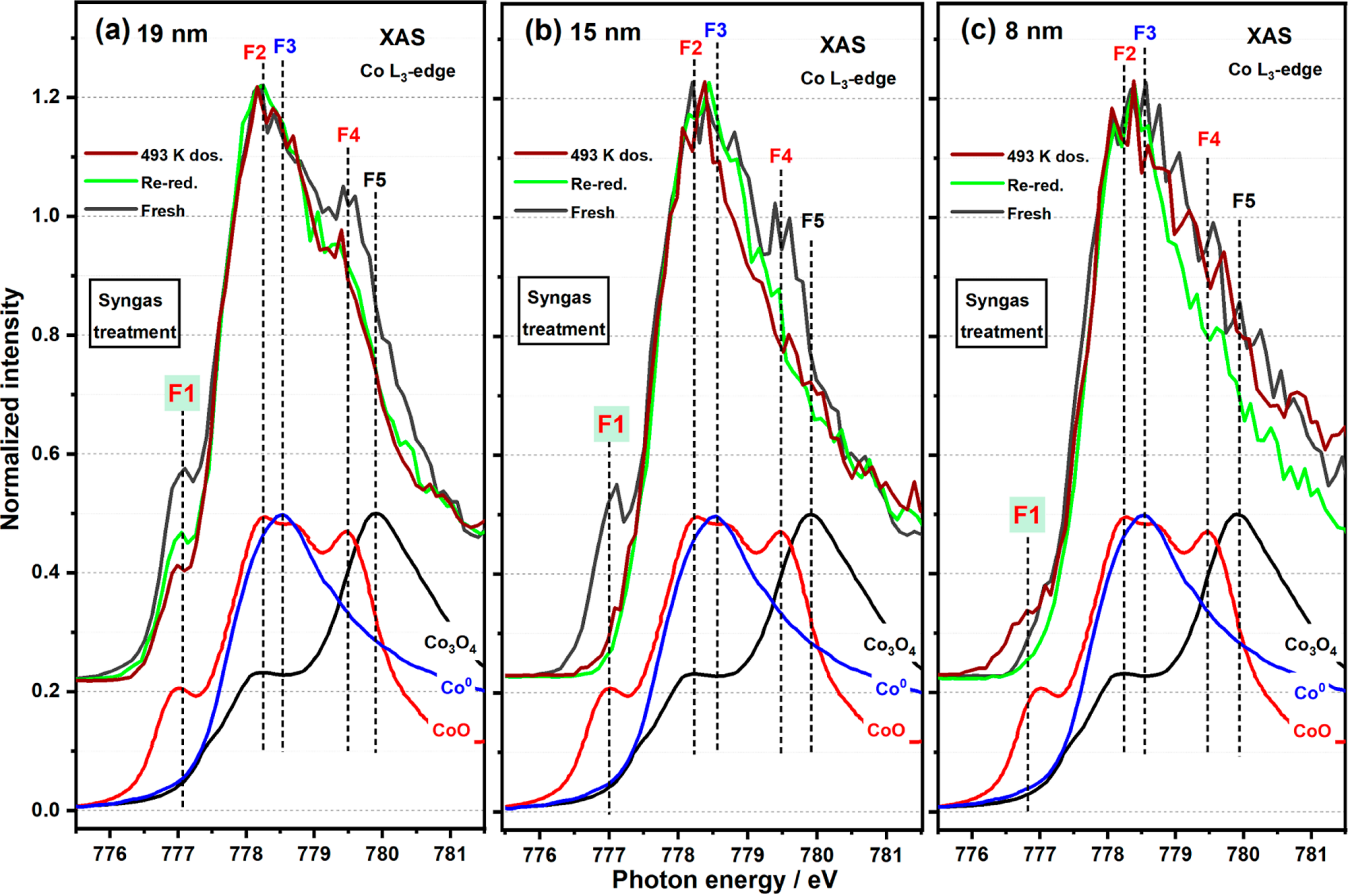
XAS spectra of Co L_3_-edge on 19, 15, and 8 nm CoNPs under syngas treatment. Syngas dosing at 493 K promoted further reduction of cobalt oxide (e.g., 19 nm) while reoxidation in small NPs (e.g., 8 nm).

Before being exposed to syngas, the sample was re-reduced in 623 K H_2_ (1 × 10^–6^ mbar) for 1 h. The bigger NPs (24/19 nm) were seen to undergo a re-reduction (Figures 6, S4, and S5) as determined by a Co L_3_-edge XAS spectrum identical to that seen for the first reduction (Figure 3b). With syngas dosing (CO/H_2_ = 2, 1 × 10^–6^ mbar, 30 min) at 493 K (but cooling down to RT in syngas before XAS measurement), small NPs (≤12 nm) were seen to oxidize as determined from the presence of an increased F1 feature in Figures 6c, S4c, and S5b,c. The extent of oxidation was observed to increase with decreasing NP size (Figure 3d).^24,28^ For the bigger NPs (>15 nm), syngas promoted further reduction (Figure 3d), as determined by the decrease in the F1 feature, clearly seen in Figures 6a, S4a and S5a. Further annealing of the sample at 493 K, 1 × 10^–9^ mbar for 30 min saw the disappearance of cobalt oxide phases in the smaller NPs and reformation of the metallic cobalt state, while for the bigger NPs, reduction was yet again further enhanced (Co^0^ increased ∼10% in 24/19 nm, Figure 3d). It has been proposed that the oxidation of the smaller NPs in the presence of syngas is due to the dissociation of CO on undercoordinated surface cobalt atoms with the assistance of H_2_.^49^

The O K-edge XAS spectra (Figures 5, S6–S8) from the NPs that underwent syngas treatment are also consistent with the above proposal. The decreased t_2g_ peak intensity in NPs ≥15 nm after syngas dosing indicates that syngas can easily remove surface oxygen, promote the formation of new O_vac_, and subsequently facilitate the reduction of cobalt oxide. This is also confirmed with Ti L-edge XAS spectra in Figure S9. For NPs ≤12 nm, syngas dosing leads to an increase in the t_2g_ intensity (Figures 5c and S8b,c), and this increased t_2g_ peak intensity demonstrates that the O_vac_ are diminished by accepting the dissociated O* from CO promoted by H_2_. Subsequently, the dissociated O* strongly binds with surface cobalt and becomes difficult to remove, causing reoxidation of the NPs. Furthermore, the O* also diffuses onto the vicinal TiO_2_ to cause the O_vac_ loss. Either way, the t_2g_ peak intensity is seen to increase in the small NPs. This diffusion is enhanced with sample annealing, leading to cobalt oxide reduction caused by filling of the O_vac_ (Figures S6 and S7 brown spectra). We propose, however, that the formation of cobalt oxide (CoO) in small NPs is due to promoted CO dissociation in syngas. However, due to the promotion of vicinal O_vac_ (capturing O from the NPs) and the presence of inherent cobalt oxide, it is difficult to determine whether CO dissociation can also occur on larger NPs. We propose that the formation of unreducible cobalt titanate (CoTiO_3_) is not present in the sample, as small NPs can be fully reduced to metallic Co^0^ under the comparatively mild conditions applied here.^9,56^

**Figure 7:**
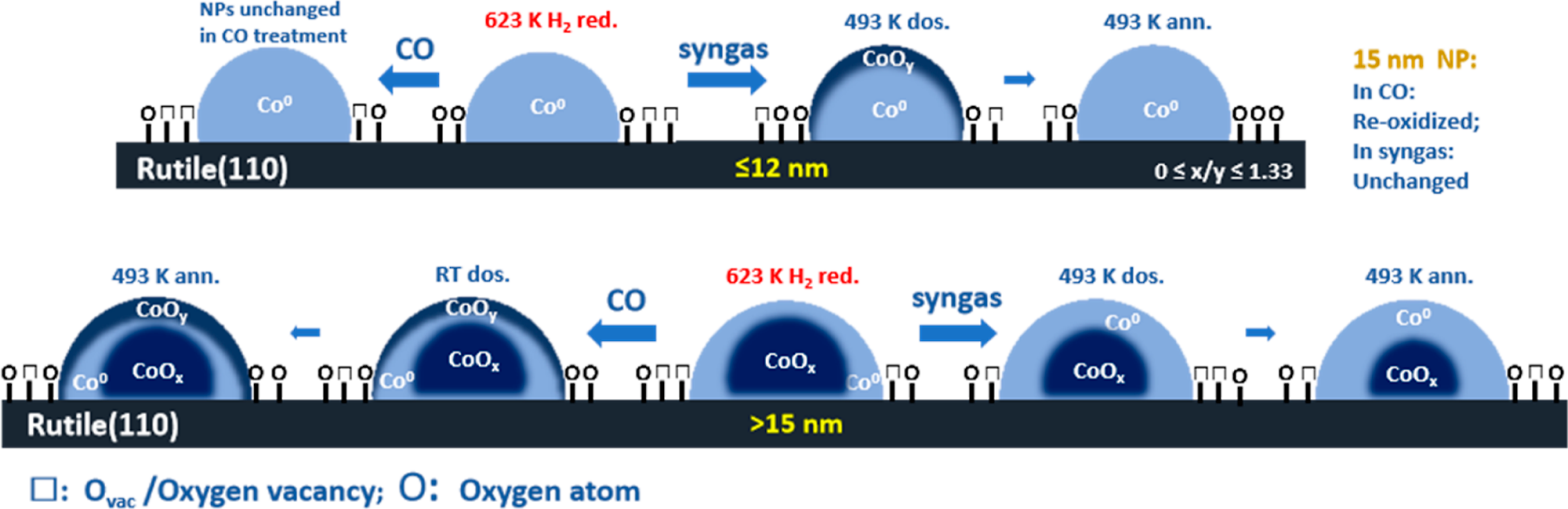
Schematic of CoNP changes during the CO/syngas treatment. In (a) small NPs are fully reduced in H_2_ (≤15 nm) but oxidized in syngas (≤12 nm). In comparison (b), bigger NPs (≥15 nm) are oxidized in CO but reduced in syngas.

The compositional evolution of the NPs is depicted in Figure 7 and is rationalized as follows. Many reports state that small CoNPs are easily oxidized by the side-product of water in FTS.^11,57^ Wolf et al.^24,58^ observed that CO dissociation was a direct factor for CoNP oxidation but that water would prevent removal of surface dissociated oxygen on a Co/SiO_2_ catalyst. This oxidation is particularly noticeable in small NPs (<5.3 nm, with a high proportion of exposed undercoordinated atoms) and the cobalt-support interface (forming cobalt-support compounds, e.g., cobalt silicate) in accordance with thermodynamic predictions.^24^ Tuxen and coauthors^49^ reported that CO dissociation on small CoNPs (4 nm) was minimal at either RT or 523 K without the assistance of H_2_, while on bigger NPs (15 nm), CO itself could directly dissociate at RT and this becomes enhanced at 523 K; thus, the surface of larger NPs become oxidized (Figure 7b). Adding H_2_ not only promotes CO dissociation on all sizes of NPs but also leads to the desorption of O anions and the regeneration or maintenance of CoNPs in the metallic state.^49^ Size-dependent CO dissociation can be further explained by the increased number of step-edge (B5) sites when increasing the size of NPs.^59^ In comparison to terrace sites, step-edge sites are considered highly active for direct CO dissociation without the assistance of H_2_.^60^

CoNPs in our case exhibit similar behavior in CO and syngas to that reported in previous work, namely, CO tends to dissociate on big NPs (>12 nm) at RT, although this is enhanced at 493 K, whereas syngas treatment promotes CO dissociation on all sizes of NPs, only in the case of the small NPs (≤12 nm) is oxidation observed. Further reduction of cobalt oxide in NPs >15 nm is detected during syngas dosing and proceeds further during annealing at 493 K. Wolf and co-workers proposed that dissociated O (from CO) strongly binds with undercoordinated sites on small NPs and is difficult to remove despite continuous syngas treatment (Figure 7a). In contrast, the binding of O on bigger NPs is proposed to be weak, and thus surface O can be easily removed by H_2_ in syngas. The surface containing adsorbed O species on small NPs is shown to be metastable, with these species being able to be easily removed during UHV annealing and leading to the reformation of metallic cobalt (Figure 7a).^25^ This CO dissociation (with or without H_2_) not only leads to the reoxidation of NPs but also affects surface O_vac_ on TiO_2_ which, we have already shown, influences the stability of CoNPs by capturing any vicinal oxygen-containing compounds.^61^ These results also demonstrate that the reduction of cobalt oxides in syngas is more facile than when using pure H_2_; taking the data for the bigger NPs (24 and 19 nm) as exemplars, the reducibility is significantly improved after syngas treatment (Figure 3d). This further reduction has been observed in previous work^26^ and could be due to the lower Gibbs free energy for surface O removal in the presence of CO,^62^ implying the important role of CO or joint effect of CO and H_2_ for promoting cobalt oxide reduction.

## 3 Summary and Conclusions

The aim of this work was to understand the component evolution of individual CoNPs with different sizes in CO/syngas treatment by using a spatially resolved technique of XAS/X-PEEM. To this end, a 2D Co/TiO_2_ catalyst supported with individual CoNPs (6–24 nm) on a rutile (110) substrate was prepared. NPs smaller than 15 nm appeared notably reduced in the fresh sample, likely as a result of the presence of O_vac_ on TiO_2_ and these NPs subsequently become fully reduced to cobalt metal in 623 K H_2_. It was determined that pure CO cannot dissociate on small CoNPs (<15 nm). However, strong dissociation of adsorbed CO does occur on big NPs (>15 nm) leading to them being reoxidized, although this is mitigated by the diminishing of vicinal O_vac_ even at room temperature. Reoxidation is seen in NPs ≤12 nm during the subsequent syngas adsorption (at 493 K) while further reduction of cobalt oxide is observed in big NPs (>15 nm). The presence of H_2_ is thought to promote CO dissociation and strongly bind on small NPs, although the reoxidized small NPs are unstable and can be regenerated to form metallic cobalt following 493 K UHV annealing. However, the combined effect of H_2_ and CO in syngas promoted the easy removal of oxygen in big CoNPs (>15 nm) resulting in the production of more metallic cobalt. Indeed, such observations are consistent with previous reports showing that oxidized CoNPs tend to reduce to metallic cobalt under FTS operating conditions, as previously reported for Co/Al_2_O_3_ catalysts.^26^ However, in this study, the extent of reduction of CoNPs was particularly affected by the presence of O_vac_, particularly vicinal O_vac_, promoting the removal of adsorbed O/presence of oxidized Co on the NPs, returning them to the reduced state. Syngas and CO were shown to be particularly capable of inducing the formation of O_vac_ on TiO_2_ and suggested an additional indirect process by which CoNPs are retained in the metallic state understood to be necessary for FTS activity. This suggested a particular advantage of using TiO_2_ instead of nonreducible supports such as SiO_2_ and Al_2_O_3_ which may explain its popularity as a support for industrial FTS. Furthermore, since we observe a size dependency of the CoNP composition during CO/syngas treatment, it is possible to use this information to prepare catalysts with specific particle sizes and to understand how these behave during activation in different reductive atmospheres and ultimately how better FTS performance and stability can be achieved.

## 4 Methods

### 4.1 Catalyst Preparation

#### 4.1.1 Co_3_O_4_ NP Synthesis

2 g of tetraethylene glycol monododecyl (C_12_E_4_, Brij L4, Sigma-Aldrich) mixed with 10.67 g of *n*-hexane (Sigma-Aldrich) was put into a 301 K water bath and stirred at 500 rpm for 2 h to form a reverse micelles solution. Then, 384 mg cobalt nitrate hexahydrate (Sigma-Aldrich) in 0.4 mL DI water was added and kept stirring for another 2 h under the same conditions. After that, 25 wt % NH_3_ (aq) (0.9 g, Sigma-Aldrich) was added to generate Co(OH)_2_ NPs, and the solution was kept stirring for 1 h. >60 mL acetone was added to break micelles and release Co(OH)_2_ NPs. Moreover, the NPs were continually washed 3–5 times using acetone to remove C_12_E_4_ before drying at 393 K for 12 h and calcining at 473 K for 5 h.^63^ The generated Co_3_O_4_ NPs were shown in Figure S1b. Varying the amount of cobalt nitrate being added, the size of Co_3_O_4_ NPs could be modulated (shown in Figure S1a).

#### 4.1.2 2D Cobalt Catalyst Preparation

The rutile TiO_2_ (110) substrate (10 × 5 × 1 mm, GmbH) was calcined at 773 K for 6 h in a muffle furnace and then cleaned in an ultrasonic bath with acetone and isopropanol. Co_3_O_4_ NPs (mixtures of 6, 11, and 18 nm NPs) were dispersed into 10 mL of ethanol in an ultrasonic bath (20 min). Then upon removing some agglomerated NPs following centrifugation (8000 rpm, 5 min), the solution became transparently yellow. NP dip-coating onto the substrate was performed at room temperature with a draw speed of 5 mm/min. After mild calcination (473 K, 5 h), the prepared sample was further treated in air plasma (0.3 mbar, 100 w, 1 h).

### 4.2 Scanning Electron Microscopy

To correlate the real size of focused Co NPs in the X-PEEM images, the samples after X-PEEM measurement were imaged by a Carl Zeiss crossbeam 550 scanning electron microscope (EHT = 2.0/5.0 kV). The images were analyzed by using the ImageJ 1.52e software.^64,65^

### 4.3 X-Ray Diffraction

A Rigaku SmartLab X-ray diffraction (XRD) instrument (Cu Kα1, 45 kV, 2θ 20–70°, step 0.01°, speed 0.2 s/°) with fixed divergence slits at ISIS neutron and muon light source was used to measure the prepared NPs. The average nanoparticle size was estimated by the Scherrer equation using the ⟨311⟩ facet as the NPs were confirmed to be Co_3_O_4_.

### 4.4 X-Ray Photoelectron Spectroscopy

X-ray photoelectron spectroscopy (XPS) analysis for the sample was performed on a Thermo Fisher Scientific NEXSA spectrometer at HarwellXPS. This spectrometer was equipped with a microfocused monochromatic Al X-ray source (72 W, 400 μm). Data were recorded at pass energies of 50 eV for Co 2p and O 1s scans with a 0.1 eV step size. The sample was measured under a vacuum of 10^–9^ mbar and at room temperature with a charge neutralization mode. The recorded data were analyzed by CasaXPS (version 2.3.19PR1.0).^66^ The binding energy was calibrated using C 1s (284.8 eV).

### 4.5 X-Ray Photoemission Electron Microscopy

X-PEEM was carried out at I06 at DLS with a high-brilliance X-ray light in the energy range of 130–1500 eV. The elemental contrast X-PEEM images (field of view 6 μm) were recorded at the cobalt L_3,2_-edge absorption edge by using a total electron yield (TEY) mode. The bright spots may correspond to individual Co NPs but have to be confirmed by XAS. The base pressure in the X-PEEM was 1 × 10^–9^ mbar and annealing of the samples was started in this condition. The XAS spectra of Co L_3,2_-edge, O K edge, Ti L_3,2_-edge, and C K-edge were recorded at the same conditions. Dosages of hydrogen, carbon monoxide, and syngas were controlled at 1 × 10^–6^ mbar in a prechamber. The gas treatment on the sample was controlled at different conditions: hydrogen reduction (623 K, 3 h); carbon monoxide adsorption (room temperature/623 K, 30 min); and syngas adsorption (493 K, 30 min). Sample annealing was conducted in the analysis chamber without a gas atmosphere (1 × 10^–9^ mbar, 493 K, 30 min). The X-PEEM images were processed by the ImageJ 1.52e software, while the XAS spectra were analyzed by the Origin Pro 2019. All of the spectra of the Co L_3_-edge below 776 eV were smoothed and subtracted by the TiO_2_ background. The linear combination fitting for the Co L_3_-edge (775–784 eV) was done by using Athena 0.9.26 software.

## Supporting Information Available

Additional sample characterization data including XRD; supplementary soft X-ray spectra; and exemplar fits to the XAS data (PDF)
