# Supplementary material for: Compositional Evolution of Individual CoNPs on Co/TiO2 during CO and Syngas Treatment Resolved through Soft XAS/X-PEEM
Source: ACS Catal. 2023 Nov 28;13(24):15956–66. doi: 10.1021/acscatal.3c03214 (PMC10729030; doi:10.1021/acscatal.3c03214)
Supplement: Supplementary file 1 — cs3c03214_si_001.pdf [file cs3c03214_si_001.pdf]

Supporting information

Compositional evolution of individual CoNPs on Co/TiO_2 during CO and syngas treatment resolved through soft XAS/X-PEEM

Chengwu Qiu[1, 2], Yaroslav Odarchenko[1, 2], Qingwei Meng[3], Hongyang Dong[1, 2], Ines Lezcano Gonzalez[1, 2], Monik Panchal[1, 2], Paul Olalde-Velasco[4 #], Francesco Maccherozzi[4], Laura Zanetti-Domingues[2], Marisa L. Martin-Fernandez[2], Andrew M. Beale[1, 2 *]

[1]Department of Chemistry, University College London, 20 Gordon Street, London, WC1H 0AJ, UK

[2]Research Complex at Harwell (RCaH), Harwell, Didcot, Oxfordshire, OX11 0FA, UK

[3]School of Chemical Engineering and Light Industry, Guangdong University of Technology, Guangzhou, 510006 (China)

[4]Diamond Light Source, Harwell, Didcot, Oxfordshire, OX11 0DE, UK

**Corresponding author: Andrew.Beale@ucl.ac.uk*

#Departamento de Ciencias Quimicas, Facultad de Estudios Superiores Cuautitlan, Universidad Nacional Autonoma de Mexico, Av. Primero de Mayo s/n, Cuautitlan Izcalli, Estado de Mexico 54740, Mexico

Table of contents

Figure S1. XRD patterns of Co_3O_4 NPs used for Co/TiO_2 preparation.

Table S1. Linear combination fitting results of XAS spectra of Co L_3-edge (775-784 eV) in treatments.

Figure S2. Examples of linear combination fitting results for Co L_3-edge.

Figure S3. XAS spectra of O K edge and XPS spectra of O 1s and Ti 2p in fresh Co/TiO_2 catalyst.

Figure S4. XAS Co L_3-edge spectra of 19, 15 and 8 nm Co NPs in CO/syngas treatment.

Figure S5. XAS Co L_3-edge spectra of 24, 12 and 6 nm Co NPs in CO/syngas treatment.

Figure S6. Local XAS O K-edge spectra of 19, 15 and 8 nm Co NPs in CO/syngas treatment.

Figure S7. Local XAS O K-edge spectra of 24, 12 and 6 nm Co NPs in CO/syngas treatment.

Figure S8. Local XAS O K-edge spectra of 24, 12 and 6 nm Co NPs after CO/syngas dosing.

Figure S9. XAS spectra of Ti L edge of 24, 19, 15, 12, 8 and 6 nm NPs during various treatments.

Figure S10. XAS spectra of C K edge on the 24, 19, 15, 12, 8 and 6 nm NPs during various treatments.

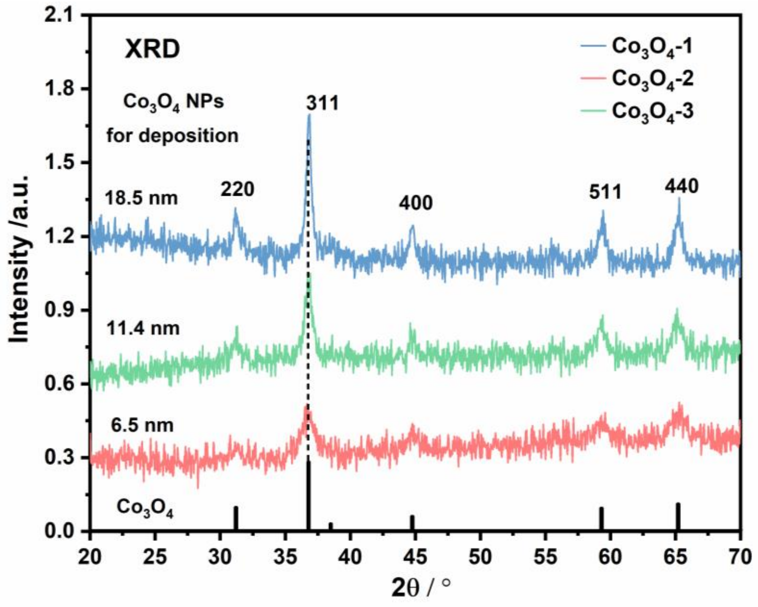

Figure S1. XRD patterns of Co_3O_4 NPs used for Co/TiO_2 preparation. The Co_3O_4 nanoparticles used for sample preparation are the mixtures of Co_3O_4-1, Co_3O_4-2 and Co_3O_4-3.

Table S1. Linear combination fitting results of XAS spectra of Co L_3-edge (775-784 eV) in treatments.

	NP/nm	Co^0/%	CoO/%	Co_3O_4/%	R-factor	Reduced Chi-square
Fresh	24	10.2(3.0)	54.6(2.1)	35.2(2.3)	0.02626	0.003201
	19	31.8(3.2)	46.2(3.2)	22.0(4.6)	0.01616	0.001549
	15	59.1(4.0)	24.6(2.8)	15.3(0.3)	0.01293	0.001149
	12	64.7(7.1)	20.9(5.7)	14.4(9.7)	0.06659	0.005308
	8	98.3(5.9)	1.5(5.9)	0.2(8.4)	0.05774	0.005182
	6	100(0)	0(0)	0(0.8)	0.06450	0.005417
Reduction	24	37.9(5.6)	62.1(4.0)	0(0.8)	0.01460	0.001991
	19	71.6(3.0)	28.4(3.2)	0(1.2)	0.01021	0.000931
	15	100(0)	0(0)	0(0)	0.00808	0.000678
	12	100(0)	0(0)	0(0)	0.01168	0.001020
	8	100(0)	0(0)	0(0)	0.02734	0.002445
	6	100(0)	0(0)	0(0)	0.02309	0.001851
CO dosing	24	36.5(4.1)	61.1(4.1)	2.4(6.1)	0.02470	0.002409
	19	62.6(3.5)	32.1(4.5)	5.3(1.4)	0.01360	0.001258
	15	96.6(0.9)	3.4(1.9)	0(0.6)	0.02331	0.001817
	12	100(0)	0(0)	0(0)	0.02518	0.001973
	8	100(0)	0(0)	0(0)	0.04113	0.002879
	6	100(0)	0(0)	0(0)	0.06043	0.004818
UHV ann. after CO dos.	24	26.5(4.8)	73.5(3.3)	0(1.6)	0.01526	0.001559
	19	59.6(7.3)	39.8(5.0)	0.6(2.3)	0.04128	0.003570
	15	92.0(3.4)	7.6(2.9)	0.4(4.4)	0.01452	0.001202
	12	99.9(7.9)	0.1(6.3)	0(0.1)	0.07702	0.005845
	8	100(0)	0(0)	0(2.2)	0.04299	0.003369
	6	100(6.6)	0(4.1)	0(1.9)	0.03313	0.002459
Re-reduction	24	39.7(4.6)	60.3(3.2)	0(0.4)	0.01501	0.001471
	19	67.7(4.4)	32.3(2.8)	0(1.3)	0.01269	0.001107
	15	100(3.0)	0(2.7)	0(1.2)	0.01331	0.001084
	12	100(0)	0(0)	0(3.9)	0.02759	0.002326
	8	100(3.7)	0(3.7)	0(0.5)	0.02487	0.002014
	6	100(0)	0(0)	0(3.9)	0.01659	0.001339
Syngas dosing	24	44.5(4.9)	54.8(5.0)	0.7(1.1)	0.03217	0.003241
	19	74.4(3.3)	25.6(2.4)	0(0.5)	0.01845	0.000831
	15	100(4.7)	0(4.4)	0(1.7)	0.04132	0.002380
	12	95.2(6.6)	0(6.3)	4.8(2.1)	0.08219	0.005872
	8	91.9(10.4)	1.2(6.9)	7.0(2.4)	0.09650	0.007012
	6	87.7(10.8)	12.3(7.7)	0(0.2)	0.01068	0.008848
UHV ann. after syngas dos.	24	47.5(6.9)	52.5(4.3)	0(2.0)	0.02684	0.002619
	19	80.3(4.8)	19.7(3.3)	0(0.4)	0.01874	0.001630
	15	100(0)	0(0)	0(0.3)	0.03647	0.002562
	12	100(0)	0(0)	0(0.4)	0.04421	0.004352
	8	100(0)	0(0)	0(0.5)	0.01870	0.003054
	6	100(0)	0(0)	0(0.4)	0.04889	0.004302

Note. Values in brackets represent uncertainties in the amount of each Co component in the nanoparticle.

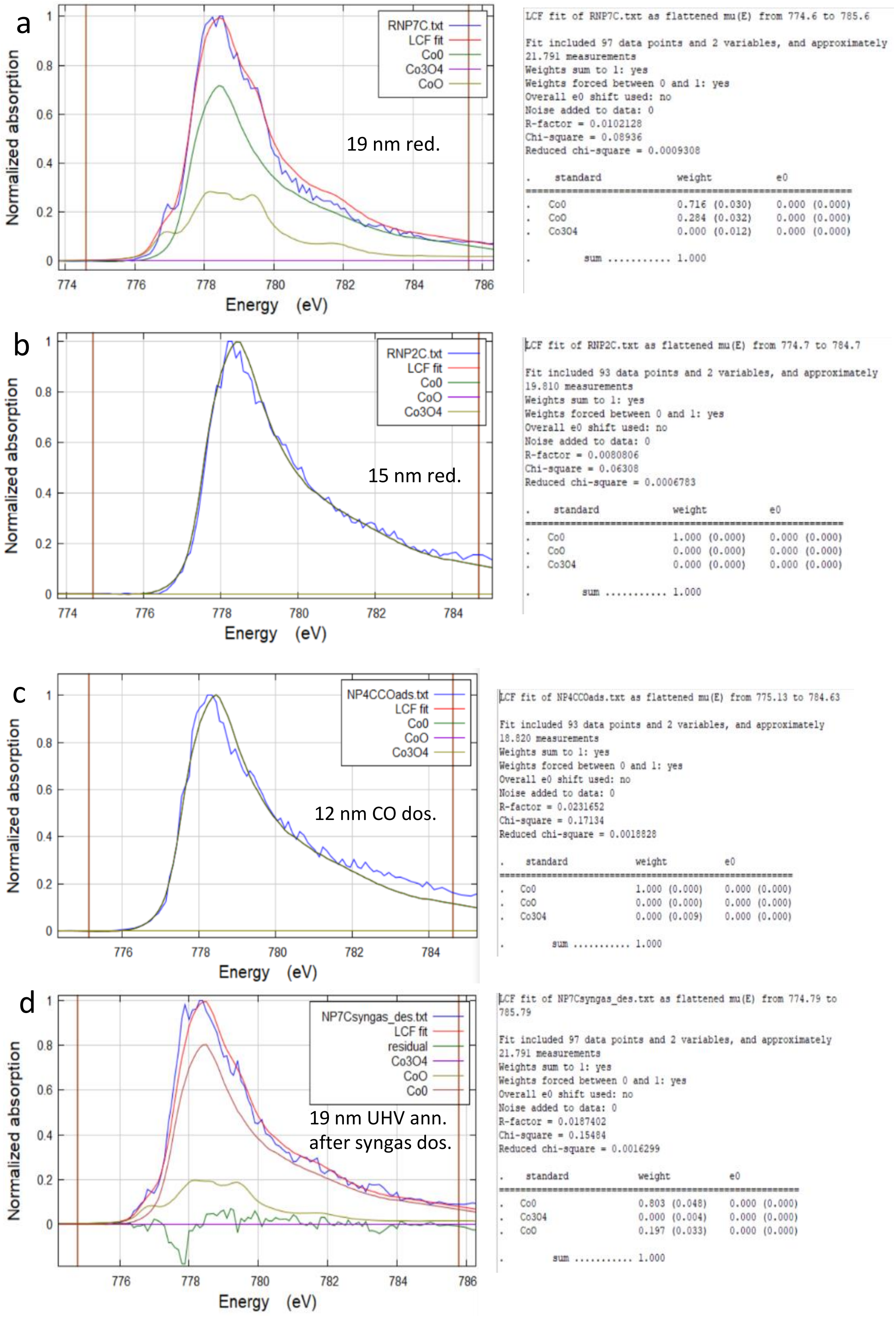

Figure S2. Examples of linear combination fitting results for Co L_3-edge.

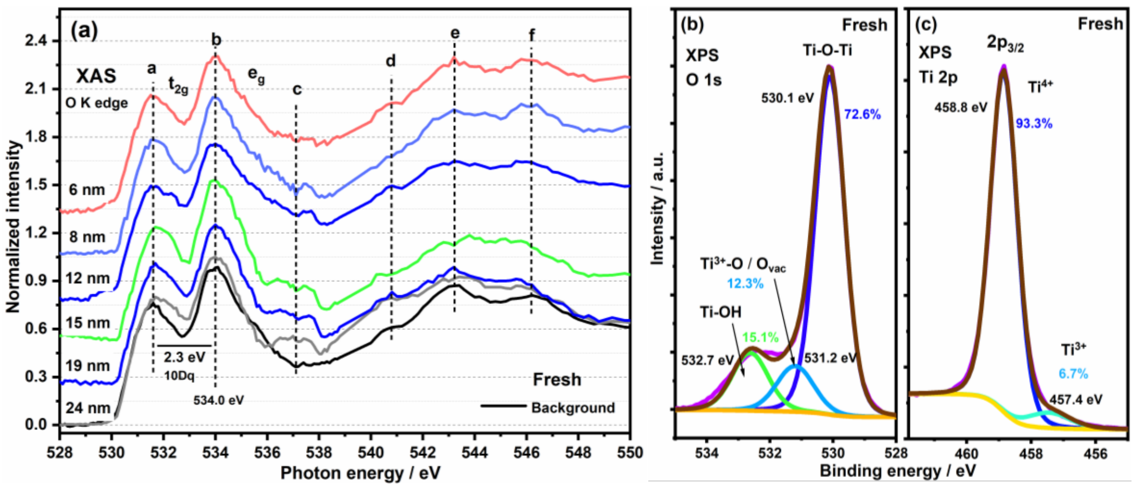

Figure S3. XAS spectra of O K edge (a) and XPS spectra of O 1s (b) and Ti 2p (c) in fresh Co/TiO_2 catalysts. All the spectra in (a) are normalized to 1 through e_g peaks.

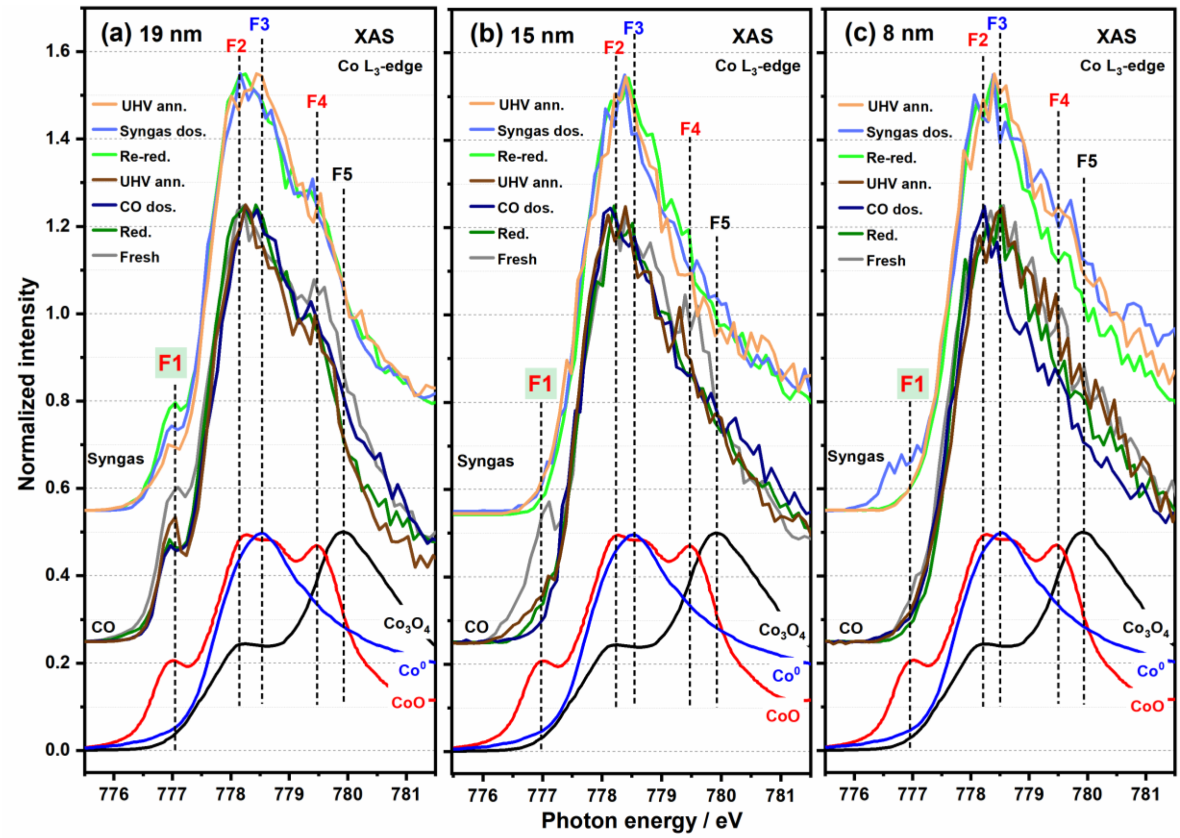

Figure S4. XAS Co L_3-edge spectra of 19, 15 and 8 nm CoNPs in CO/syngas treatment. The spectra were recorded in the centre of the NPs. Big NPs (≥ 15 nm) were re-oxidised due to a greater tendency of CO to dissociate.[5] Syngas adsorption at 493 K promoted cobalt oxide reduction in larger NPs whilst re-oxidation in small NPs (i.e. 8 nm) was observed. However, re-oxidised small NPs were unstable and NPs could re-reduce during isothermal annealing, thought to be promoted by surface oxygen vacancies on the TiO_2.

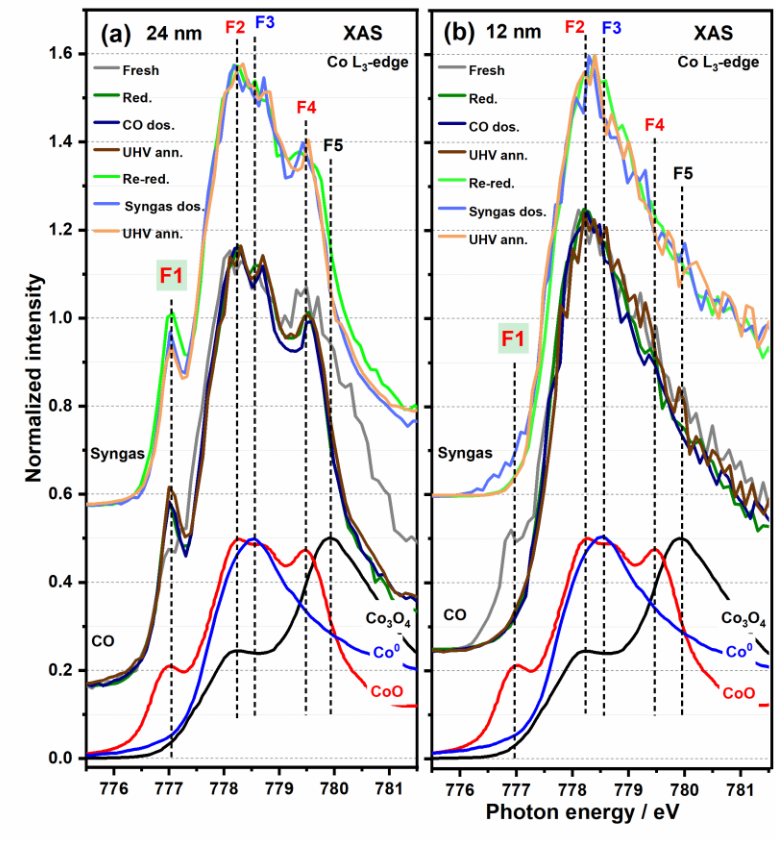

Figure S5. XAS Co L_3-edge spectra of 24, 12 and 6 nm CoNPs in CO/syngas treatment. The spectra were recorded in the centre of the NPs.

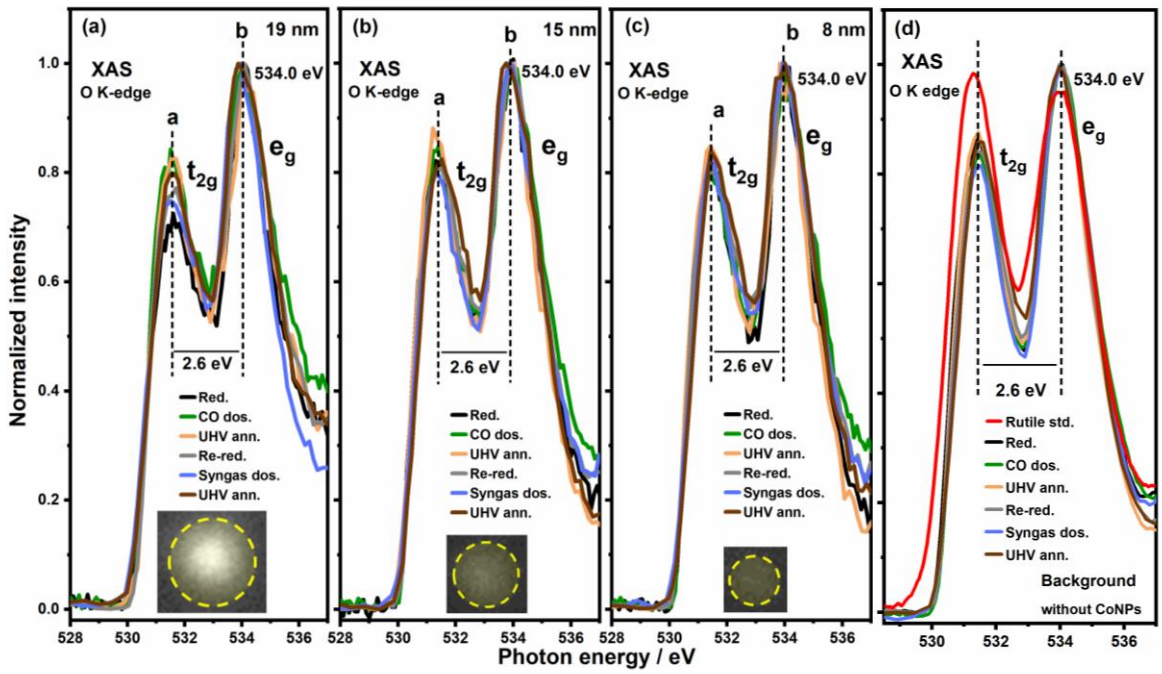

Figure S6. XAS spectra of O K-edge on 19, 15, 8 nm NPs and background titania after gas dosing in Co/TiO_2. The spectra were recorded from within the yellow dashed line around the different-size NPs as indicated above. All the spectra are normalized to 1 through e_g peaks. The t_{2g} intensities of O K-edge lower than e_g as well as their low splitting energies (10Dq = 2.7 eV in standard rutile) mean presenting of O_{vac}.

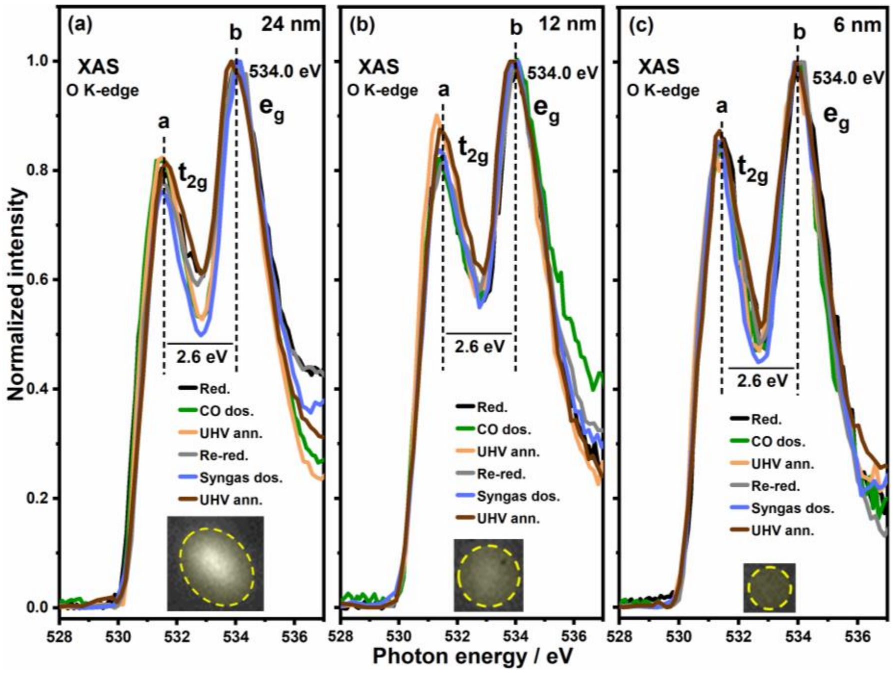

Figure S7. Local XAS O K-edge spectra of 24, 12 and 6 nm CoNPs in CO/syngas treatment. The spectra were recorded from the NPs (differing in size) as indicated with a yellow ring. All the spectra are normalized to 1 through e_g peaks.

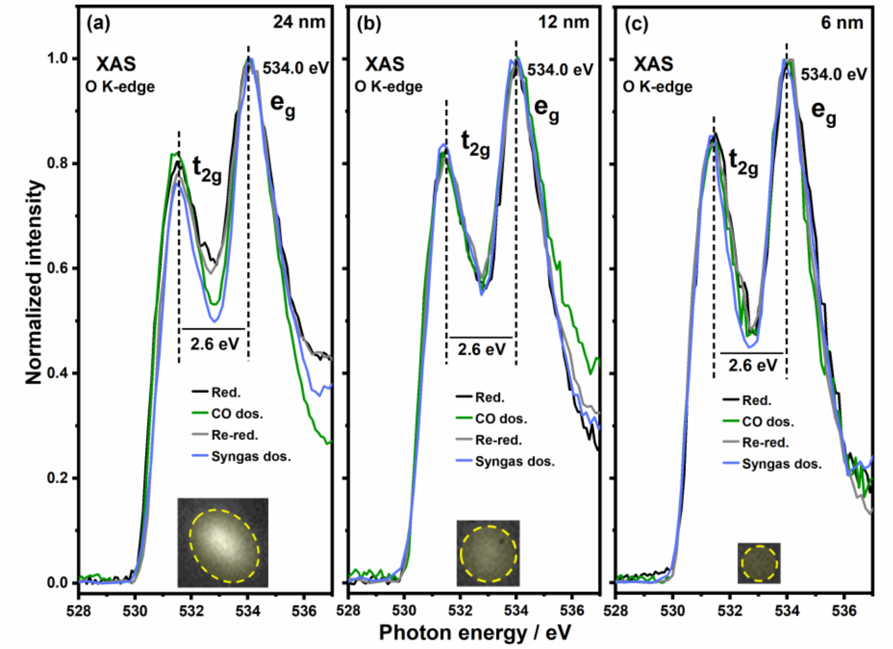

Figure S8. Local XAS O K-edge spectra of 24, 12 and 6 nm Co NPs after CO/syngas dosing. All the spectra are normalized to 1 through e_g peaks.

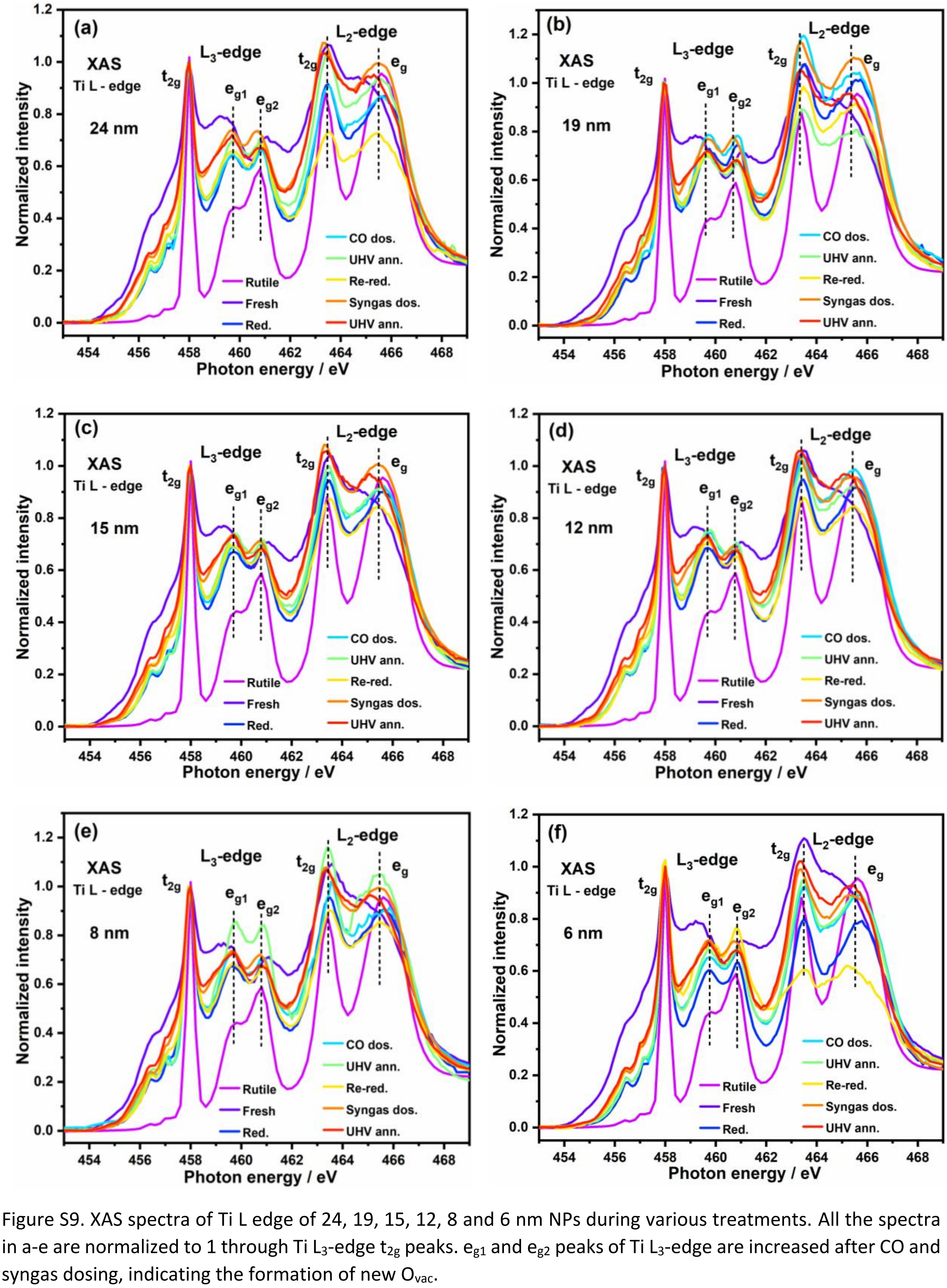

Figure S9. XAS spectra of Ti L edge of 24, 19, 15, 12, 8 and 6 nm NPs during various treatments. All the spectra in a-e are normalized to 1 through Ti L_3-edge t_{2g} peaks. e_{g1} and e_{g2} peaks of Ti L_3-edge are increased after CO and syngas dosing, indicating the formation of new O_{vac}.

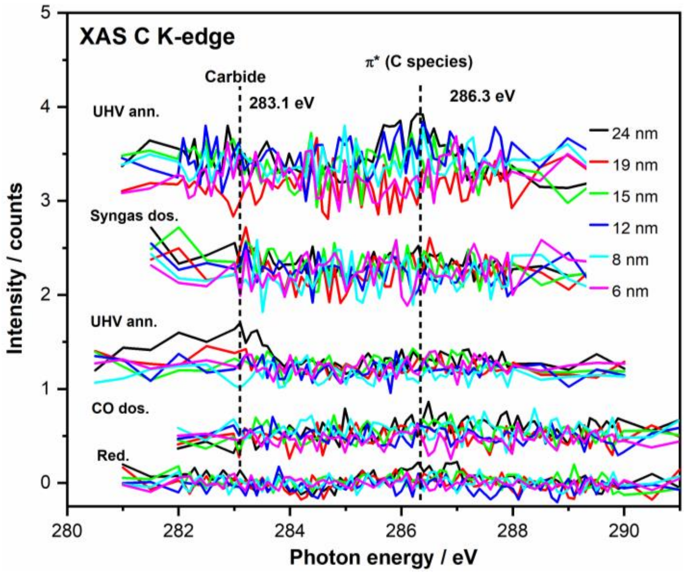

Figure S10. XAS spectra of C K edge on the 24, 19, 15, 12, 8 and 6 nm NPs during various treatments. No carbide and other changes were found for all the nanoparticles.

References

1. Fischer, N., Van Steen, E. & Claeys, M. Preparation of supported nano-sized cobalt oxide and fcc cobalt crystallites. *Catal. Today* **171**, 174–179 (2011).

2. Liu, X., Atwater, M., Wang, J. & Huo, Q. Extinction coefficient of gold nanoparticles with different sizes and different capping ligands. *Colloids Surfaces B Biointerfaces* **58**, 3–7 (2007).

3. Prathibha, V., Karthika, S., Cyriac, J., Sudarasanakumar, C. & Unnikrishnan, N. V. Synthesis of pure anatase TiO_2 nanocrystals in SiO_2 host and the determination of crystal planes by Image J. *Mater. Lett.* **65**, 664–666 (2011).

4. Biesinger, M. C. *et al.* Resolving surface chemical states in XPS analysis of first row transition metals, oxides and hydroxides: Cr, Mn, Fe, Co and Ni. *Appl. Surf. Sci.* **257**, 2717–2730 (2011).

5. Tuxen, A. *et al.* Size-dependent dissociation of carbon monoxide on cobalt nanoparticles. *J. Am. Chem. Soc.* **135**, 2273–2278 (2013).
